# Expression profiling of O^6^ methylguanine-DNA-methyl transferase in prolactinomas: a correlative study of promoter methylation and pathological features in 136 cases

**DOI:** 10.1186/s12885-015-1595-0

**Published:** 2015-09-23

**Authors:** Xiao-Bing Jiang, Bin Hu, Dong-Sheng He, Zhi-Gang Mao, Xin Wang, Bing-Bing Song, Yong-Hong Zhu, Hai-Jun Wang

**Affiliations:** 1 Department of Neurosurgery, First Affiliated Hospital of Sun Yat-sen University, Haizhu, Yixian Rd, Guangdong Guangzhou, China; 2 Key Laboratory of Pituitary Adenoma in Guangdong Province, Guangzhou, 510080 China; 3 Department of Histology and Embryology, Zhongshan School of Medicine, Sun Yat-sen University, Guangzhou, 510080 China; 4 Department of Neurosurgery, Sun Yat-sen University Cancer Center, State Key Laboratory of Oncology in South China; Collaborative Innovation Center for Cancer Medicine, Guangzhou, 510060 China

## Abstract

**Background:**

Low-level expression of O^6^ methylguanine-DNA-methyl transferase (MGMT) prolactinomas has been noted previously in case reports, although what modulates MGMT expression remains unclear. This study therefore aimed to delineate the factors regulating MGMT expression in prolactinomas.

**Methods:**

We retrospectively reviewed 136 prolactinoma patients who were treated in our center between January 2000 and September 2013. Expression of MGMT, Ki-67, and p53 protein were examined by immunohistochemical staining, and MGMT promoter methylation evaluated with methylation-specific PCR.

**Results:**

MGMT immunopositivity was <25 % in 106/136 tumor specimens (77.94 %). MGMT immunoexpression was positively correlated with age (*r* = 0.251, *p* = 0.003), but inversely correlated with p53 staining (*r* = −0.153, *p* = 0.021). Moreover, reduced MGMT expression was more frequent in atypical prolactinomas (*p* = 0.044). Methylated MGMT promoter was confirmed in 10/46 specimens (21.7 %), all of which had low level or absent MGMT staining. Both p53 protein (*r* = −0.33, *p* = 0.025) and promoter methylation (*r* = −0.331, *p* = 0.025) were negatively associated with MGMT expression. Multivariate logistic analysis indicated that age (odds ratio [OR] = 1.127. 95 % confidence interval [CI] 1.027–1.236, *p* = 0.012) and p53 (OR = 0.116. 95 % CI 0.018–0.761, *p* = 0.025) staining were independent determents of MGMT expression.

**Conclusions:**

The majority of prolactinomas, especially atypical prolactinomas, showed low-level or no MGMT immunoexpression, providing a rationale for the utility of temozolomide as an alternative to managing prolactinomas. In summary, epigenetic and transcriptional regulation are involved in silencing MGMT expression.

## Background

Prolactinomas are among the most prevalent secretory pituitary adenomas and are usually controlled by dopamine agonists, with or without adjuvant surgical and radiation therapy [1]. However, there are limited treatment options for patients who harbor drug-resistant prolactinomas, where surgical resection either failed or early recurrence occurred after surgery or radiotherapy. Temozolomide (TMZ) is an oral alkylating agent and has shown promise against glioblastoma [2]. More recently, successful use of TMZ in treating aggressive pituitary adenomas and pituitary carcinomas has been widely reported [3]. However, the mechanism by which TMZ acts on pituitary tumors remains unclear. Previous studies have documented that O^6^ methylguanine-DNA-methyl transferase (MGMT) correlates with the response of glioblastomas to TMZ [4, 5]. Similarly, low-level MGMT is also associated with a better prognosis in TMZ-treated adenoma patients [6]. Interestingly, prolactinomas are more sensitive to TMZ, with 75 % of published cases showing a sustained response [5]. However, accumulating evidence indicates that low-level MGMT is more frequently observed in prolactinomas [5, 7, 8], suggesting a potential association between responsiveness and reduced MGMT expression [5]. Despite the fact that MGMT expression might help predict prolactinoma response to TMZ, to our knowledge, the expression profile of MGMT in prolactinomas has not been systematically described.

The mechanisms underlying the genetic, epigenetic, and transcriptional regulation of MGMT expression are not fully understood [9–11]. Promoter methylation represents one of the major factors silencing MGMT gene expression and predicts a favorable outcome in patients with glioblastomas exposed to TMZ [2]. However, systematic studies have identified a role for MGMT promoter methylation in silencing among pituitary adenomas [5, 6, 12]. Notably, recent data implies that promoter methylation is not the leading mechanism contributing to low pituitary adenoma MGMT expression [5, 7]. At the same time, methylated MGMT promoters did not tend to correlate with a response to TMZ exposure [5]. Moreover, although the majority of sporadic pituitary adenomas are monoclonal, MGMT staining was vastly different between tumors, which again suggested that transcriptional modifications are involved in regulating MGMT expression [12–14].

The role of p53 in modulating MGMT expression is controversial. Several studies have suggested an inverse correlation of p53 with MGMT expression [15, 16]. However, it has also been shown that accumulation of p53 protein suppresses MGMT expression and promotes cell sensitivity to alkylating agents [17–20]. Furthermore, wild-type p53 protein abrogates MGMT expression by binding directly to the MGMT promoter or by sequestering specificity protein 1 (sp1) [9]. Interestingly, p53 appears to stimulate MGMT expression, and p53 inhibition sensitizes human glioma cells to TMZ [10, 21]. Additionally, wild-type p53, rather than mutant p53, promotes MGMT expression by modulating MGMT methylation by reducing DNMT1 expression in lung cancer cell lines [22], while transduction of IMR90 fibroblasts (human fetal lung cells) with a wild-type p53-adenoviral vector reduced MGMT expression [19]. In brief, the effect of p53 on MGMT expression may depend on its level and status in specific tissues. The reasons for these inconsistent findings remain elusive, and may be cell type dependent [20]. According to previous studies, high-level p53 is more frequent in aggressive and atypical pituitary adenomas [23], consistent with a higher incidence of low-level MGMT expression amongst more aggressive pituitary adenomas [24]. Moreover, although p53 is one of the most commonly inactivated genes in human cancer, it is rarely mutated in pituitary adenomas [25]. We therefore asked whether p53 was involved in mediating prolactinoma MGMT expression.

To address these questions, we carried out a retrospective study to evaluate MGMT expression with immunochemistry in a large prolactinoma cohort. Importantly, the potential mechanisms underpinning the suppression of MGMT expression, including MGMT promoter methylation and p53 regulation, were explored.

## Methods

### Subjects and study protocol

All prolactinoma patients in our center were prescribed bromocriptine as a first-line treatment. In line with other reports, resistance to bromocriptine administered in daily doses (15 mg) for at least 3 months was defined by an absent or poor response in normalization of prolactin (PRL) levels (normal PRL levels are less than 25 ng/mL) [26]. Surgery was considered only for patients who were drug resistant or unwilling to take drugs. By searching the tissue database of the First Affiliated Hospital of Sun Yat-sen University, all prolactinoma patients between January 2000 and September 2013 with enough paraffin-embedded tissue specimens for immunostaining were identified. We screened samples by immunostaining for growth hormone (GH), prolactin, adrenocorticotropin, follicle-stimulating hormone, luteinizing hormone, and thyroid-stimulating hormone. Only patients positive for prolactin and negative for the other pituitary hormones were enrolled, and patients treated with radiation before operation were excluded. Additionally, owing to the heterogeneity of the patient population, follow-up duration was not precise. Considering this study was not designed to address this question, follow-up information was not included in the present study. In summary, 136 specimens were included, whose clinical and imaging characteristics were reviewed, including age, sex, imaging features, and treatment modality. This study was approved by the Ethics Review Committee of the First Affiliated Hospital of Sun Yat-sen University. Written informed consent was obtained from patients for the use of their samples in this study.

Based on magnetic resonance (MR) imaging, a macroadenoma was defined by a maximal tumor diameter of >10 mm, whereas microadenomas were defined with a maximal tumor diameter of <10 mm. Invasive pituitary adenoma was defined as the presence of either cavernous sinus, supresellar, or infrasellar invasion. Cavernous sinus invasion was noted according to the Knosp classification [27]. Suprasellar invasion was defined by clear tumor growth through the diaphragm sella or above the plane of the inferior optic chiasm. Infrasellar invasion was determined by tumor growth into the sphenoid sinus or clivus. According to the 2004 World Health Organization classification, atypical adenomas were defined by their invasiveness, a MIB-1 proliferation index greater than 3 %, and an extensive nuclear staining for p53 protein [28]. Recurrence was defined by post-operative imaging studies if a neoplasm presented or tumor remnant increased in configuration over time and necessitated further interventions.

### Immunohistochemistry

Surgical tissue specimens were routinely formalin-fixed, paraffin-embedded, and sectioned at 5 μm for immunostaining using streptavidin-biotin. All slides were incubated with mouse monoclonal anti-MGMT at 1:250 dilution (MT3.1), anti-MIB-1 at 1:100 (ab66155), and rabbit polyclonal anti-p53 at 1:250 dilution (ab17990, all antibodies from Abcam, Cambridge, UK). MGMT staining was considered positive only when showing definitive staining of tumor nuclei. Internal positive controls consisted of endothelial cells. Replacement of a primary antibody with PBS served as the negative control.

Specimen immunoreactivity was assessed microscopically under ten high-powered fields (×200) by three independent observers (Hu B, Song BB, and Wang X), using a semi-quantitative method by estimating the percentage of positive tumor nuclei. Areas of positive staining within tumor nuclei and minimal background reactivity were considered representative and used for evaluating percentage immunopositivity. MGMT immunostaining was semi-quantitatively scored as 1, <10 %; 2, 10–25 %; 3, 25–50 %, and 4, <50 %, according to positive nuclear staining. Scores one and two were considered as low-level or absent MGMT expression, whereas scores three and four represent intermediate or high MGMT expression, respectively [29]. The Ki-67 index was defined as the proportion of MIB-1 positive tumor nuclei on the basis of a manual count of 500 cells. The p53 antibody recognizes the N-terminal epitope of both wild-type and mutant p53, with the p53 labeling index determined by the percentage of positively stained nuclei.

### Methylation-specific PCR

Only 46/136 tumors had sufficient tissue to allow determination of the methylation status of the MGMT promoter by methylation-specific PCR (MSP). Genomic DNA was extracted from 24 paraffin-embedded specimens and 22 frozen samples stored in-80 °C freezer according to standard protocols (TianGen DNA Mini Kit, Beijing, China). Methylation patterns were determined by bisulfite-treated modification, which converts unmethylated but not methylated cytosines to uracil. MSP was performed according to the kit protocol of DNA methylation modification (EZ DNA Methylation-Gold kit, Zymo Research, USA). Primers were specific for either methylated or the modified unmethylated DNA, as previously described [30]. DNA from normal peripheral blood lymphocytes (PBL) served as a negative control, and enzymatically methylated DNA from PBL was used as a positive control. Controls without DNA were used for each set of MSP assays. Ten microliters of each 50-μL MSP product was loaded directly onto nondenaturing 6 % polyacrylamide gels, stained with ethidium bromide, and examined under ultraviolet illumination.

### Statistical analysis

Associations between clinical, biological, imaging, or molecular features (MGMT promoter methylation, Ki-67 and p53 expression) and MGMT immunostaining were evaluated by Spearman correlation, *χ*2 test, or Fisher’s exact test. For multivariate logistic analysis, a backward elimination procedure was performed to identify independent predictors for MGMT expression. Results were expressed as odds ratios (ORs) with 95 % confidence intervals (95 % CIs). All statistical analysis was performed with SPSS 16.0 (Chicago, IL, USA). A *P* < 0.05 was considered statistically significant.

## Results

### Sample characteristics

Patients (136: 81 were female, 55 were male) were retrospectively reviewed, including 121 macroadenomas and 16 giant adenomas (>40 mm). The median age at prolactinoma diagnosis was 32 years (range, 14-75 years). Because cabergoline remains unavailable in China, bromocriptine was the only choice among the drugs for prolactinoma patients. Seventy-three patients were resistant to bromocriptine, and 38 patients were intolerant to bromocriptine as indicated by adverse sides. The medical history of the other 25 patients was unclear. The median maximum diameter of tumors was 20.5 mm (range, 5–70 mm). In addition, 60 invasive, 25 recurrent, and 21 atypical prolactinomas were identified in the present study.

### Immunohistochemistry results

**Fig. 1:**
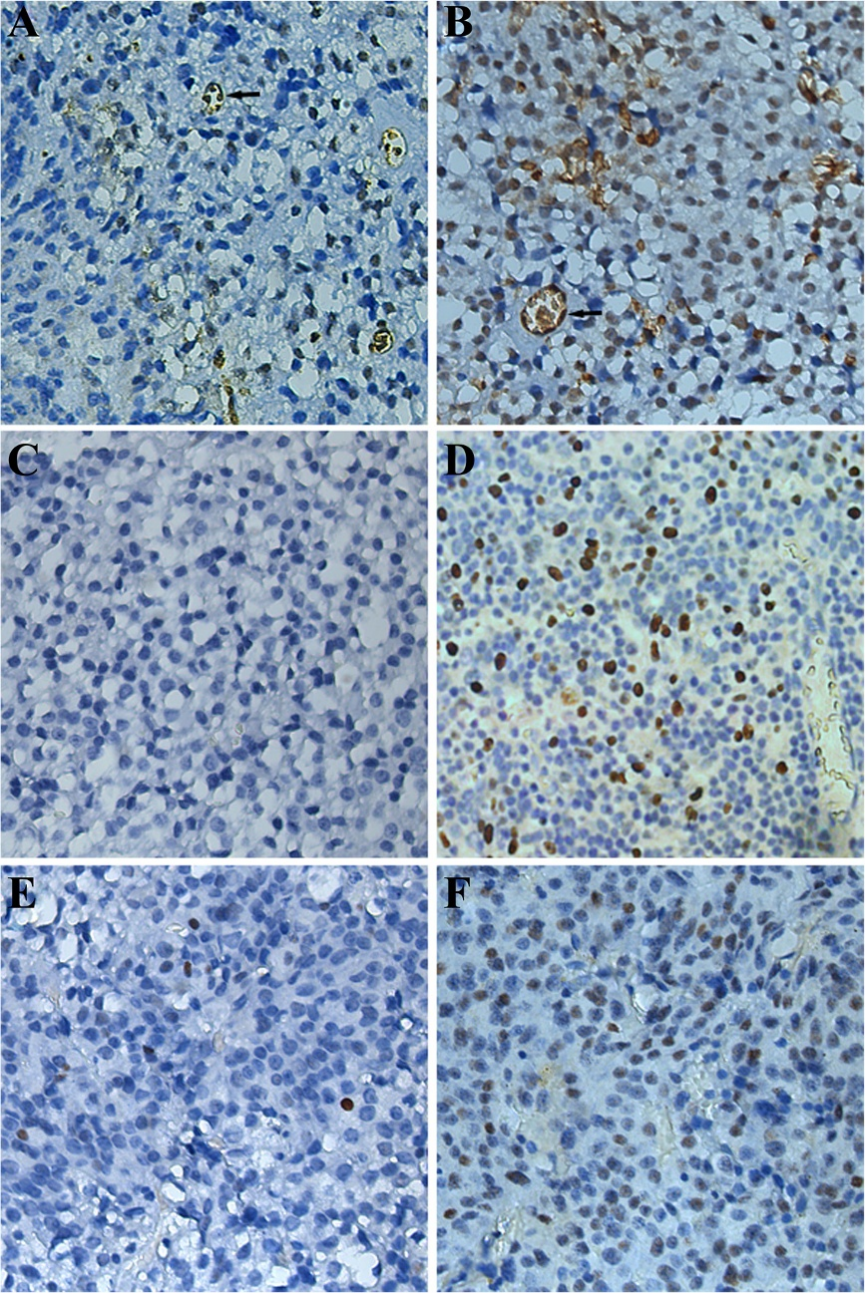
MGMT, Ki-67, and p53 protein immunohistochemistry. A prolactinoma tissue demonstrates weak (**a**) and strong (**b**) nuclear staining of MGMT. The endothelial cell (arrow) acts as an internal positive control (original magnification 400×). Negative (**c**) and positive (**d**) staining of Ki-67, and negative (**e**) and positive (**f**) staining of p53 protein are shown in prolactinoma specimens (original magnification 400×)

MGMT, Ki-67, and p53 all displayed nuclear staining (Fig. 1). Among the 136 prolactinoma specimens, MGMT immunopositivity was <10 % in 61 (44.9 %), 10–25 % in 45 (33.1 %), 25–50 % in 14 (10.3 %), and >50 % in 16 (11.8 %) of the tumors. Therefore, the majority of the specimens (78 %) showed low-level or no MGMT immunostaining. In addition, 46/60 invasive prolactinomas (76.7 %) and 18/25 recurrent species (72 %) had low-level or no MGMT expression. Fifty-seven specimens from patients resistant to bromocriptine and 29 of the intolerant patients showed low-level of no MGMT expression, respectively. There was no significant difference in MGMT expression between samples obtained from the two groups (78.08 vs. 76.32 %, *p* = 0.182).

### Both p53 and age were independent of other factors for MGMT expression

The association of MGMT expression with age, maximum diameter, p53, and Ki-67 staining were evaluated by spearman correlation analysis. Correlations with sex, recurrence, invasive, and atypical features were evaluated by *χ*2 or Fisher’s exact test. The results showed that MGMT immunoexpression was positively correlated with age (*r* = 0.251, *p* = 0.003). In contrast, there was an inverse correlation between MGMT immunoreactivity and p53 staining (*r* = −0.153, *p* = 0.021). More importantly, low-level or silenced MGMT expression was more frequently observed in atypical prolactinomas (*p* = 0.044) (Table 1). However, there were no statistically significant associations between MGMT immunostaining and sex, maximum tumor diameter, invasiveness, or recurrence (*p* > 0.05). As expected, the Ki-67 index correlated with p53 (*r* = 0.278, *p* = 0.001), tumor invasiveness (*r* = 0.241, *p* = 0.005), the maximum diameter (*r* = 0.38, *p* < 0.0001) as well as p53 staining (*p* < 0.05), but not with MGMT positive staining and recurrence (*p* > 0.05).

**Table 1:** The correlation between MGMT immuno-expression and clinical characteristics (*n* = 136)

	MGMT immuno-expression		
Variables	Low level or no expression (*n* = 106)	High expression (*n* = 30)	*P* value
Age (mean ± SD) (year)	31.34 ± 11.88	37.8 ± 11.68	0.009
Maximum diameter (mean ± SD) (mm)	22.41 ± 12.75	24.63 ± 13.61	0.408
Gender			0.369
Female	61	20	
Male	45	10	
Recurrent prolactinoma			0.428
No	88	23	
Yes	18	7	
Invasive prolactinoma			0.75
No	60	16	
Yes	46	14	
Atypical prolactinoma			0.044
No	86	29	
Yes	20	1	

Age and tumor maximum diameter were compared with the Student’s *t*-test. Categorical analysis of variables was performed using either the *χ*2 test or Fisher’s exact test, as appropriate

Seven covariates, including age, sex, recurrence, Ki-67 index, p53 staining, and the presence of atypical and invasive features, were analyzed in multiple logistic regression models. Despite the strong correlations between the Ki-67 index, p53 staining, and the presence of atypical features, they were not included in the model. Only p53 staining (OR = 0.218, 95 % confidence interval [CI]: 0.075–0.623, *p* = 0.005) and age (OR = 1.048, 95 % confidence interval [CI]: 1.01–1.086, *p* = 0.021) were determined independently of the other factors for MGMT expression.

### MSP results

MSP was used to evaluate MGMT promoter methylation status in 46 samples, with methylation confirmed in 10 (21.7 %) specimens, all of which were shown to have low level MGMT staining, with seven exhibiting <10 % staining, and three with 10–25 % positive. Unmethylated MGMT promoters were detected in the remaining 36 (78.3 %) specimens, which included 23 samples with low-level MGMT expression. In the 23 specimens displaying low-level MGMT immunoexpression without a methylated MGMT promoter, 11 were positive for p53, which again indicated a potential role for p53 in regulating prolactinoma MGMT expression. By Spearman correlation analysis, both p53 protein expression (*r* = −0.33, *p* = 0.025) and methylated MGMT promoter (*r* = −0.331, *p* = 0.025) were negatively associated with MGMT expression. Because no specimen with a methylated promoter showed high-level MGMT staining, MGMT promoter methylation was not included in the logistic regression analysis. Multivariate logistical analysis showed that age (OR = 1.127. 95 % CI 1.027–1.236, *p* = 0.012) and p53 staining (OR = 0.116. 95 % CI 0.018–0.761, *p* = 0.025) were independently negatively associated with MGMT expression (Table 2).

**Table 2:** Univariate and multivariate regression analysis showing the independent determinants of the expression of MGMT (*n* = 46)

	Univariate		Multivariate	
Independent variables	OR	*P* value	OR (95 % Cls)	*P* value
Age	1.104	0.014	1.127 (1.027–1.236)	0.012
Gender	1.651	0.389		
Ki-67 index	2.321	0.237		
p53 staining	0.171	0.036	0.116 (0.018–0.761)	0.025

Except for sex and Ki-67, variables with a *P* value of <0.05 in the univariate regression analysis were included in the multivariate model. Only significant *P* values are shown in the multivariate model

## Discussion

Recently, increasing evidence suggests that TMZ might be a rational therapeutic option for patients with refractory pituitary adenomas and carcinomas [3, 5]. Surprisingly, prolactinomas appear to be more sensitive to TMZ than the other types of pituitary adenomas [5]. Meanwhile, successful TMZ therapy is closely associated with low-level MGMT expression [3, 6]. Herein, profiling MGMT expression and understanding its potential modulation in prolactinomas may pave the way for TMZ treatment of prolactinomas. Our study evaluated MGMT expression in a large cohort of atypical prolactinomas, the majority of which exhibited low-level expression. Systematic analysis showed that MGMT staining was negatively associated with its promoter methylation and p53 protein expression, indicating both epigenetic and transcriptional mechanisms are potentially involved in modulating MGMT expression in prolactinomas.

The MGMT status in pituitary adenomas has been evaluated in several previous studies by immunostaining, showing variable expression levels in different subtypes of pituitary adenomas [7, 8, 24, 31–33]. McCormack et al. first compared MGMT levels in different types of pituitary adenomas [7]. Their results showed that only 13 % of tumors (*n* = 88) demonstrated low-level MGMT expression (<10 %); however, 50 % of the prolactinomas exhibited low-level MGMT expression. Lau et al. examined MGMT expression in 30 pituitary carcinomas and 30 pituitary adenomas [29]. Low MGMT expression (<25 %) was found in 57 % of carcinomas and 60 % of invasive adenomas. Similarly, 80 % of the prolactinomas (*n* = 10) showed low-level MGMT expression. In nonfunctional pituitary adenomas, low MGMT expression (≤50 %) was confirmed in only 24 % of samples as reported by Widhalm et al. (*n* = 45) [31]. In addition, Whitelaw et al. summarized all published TMZ-treated prolactinomas, with 86.7 % staining positive at less than 10 % [5]. Thus, prolactinomas seem more likely to have reduced MGMT expression compared with other subtypes. Consistent with previous studies, 78 % of samples stained positive for low-level MGMT (25 %), with 44.9 % at less than 10 %. However, in two recent reports, low-level MGMT expression (<25 %) was observed in 91.6 % of GH adenomas (*n* = 36) [32], and 60 % of pituitary corticotroph adenomas (*n* = 40) [24]. More cases are needed to create a more accurate profile of MGMT expression in each subtype of pituitary adenoma.

MGMT expression tended to be more common in aggressive subtypes. Takeshita et al. reported 71 % of Crooke’s cell adenomas (*n* = 7) had very low MGMT expression (<5 %), whereas only 1/17 ordinary corticotroph adenomas showed low MGMT expression [34]. However, in another study, only 50 % of Crooke’s cell adenomas (*n* = 12) showed low-level MGMT staining, although all subtype I pituitary adenomas (*n* = 7) were immunopositive at <10 % [24]. Fealey et al. showed that all silent subtype 3 (SS3) pituitary adenomas (*n* = 23) had reduced MGMT expression (≤50 %), and 78 % were negative for MGMT immunoreactivity [35]. In contrast, Salehi et al. reported that 50 % of carcinomas (*n* = 10) and 92 % (*n* = 11) of SS3 pituitary adenomas showed low MGMT staining (<10 %) [12]. Similarly, we observed low-level MGMT expression more frequently in atypical prolactinomas. However, no significant relationship was found between MGMT staining and tumor size, recurrence, invasiveness, or Ki-67 index, which is similar to previous studies [3, 24, 32]. Nevertheless, these clues suggest a rational approach to administer TMZ to refractory adenomas.

In the present study, prolactinoma patients with low-level MGMT expression were younger than those with elevated expression. This is consistent with the observation that MGMT immunoreactivity was inversely correlated with patient age, again suggesting a role for methylation in regulating MGMT expression [36], although this is also likely a reflection of the younger age of the prolactinoma patients. However, no apparent relationship between MGMT expression and age was observed in patients with Cushing disease or GH adenomas [24, 32], which might be because of the various mechanisms of MGMT expression in different adenoma subtypes.

The precise mechanisms responsible for MGMT expression remain poorly understood. MGMT promoter methylation, well known as one of the proposed mechanisms underlying suppression of MGMT expression, is associated with the reduced MGMT protein level commonly seen in primary human neoplasms [37]. Moreover, MGMT promoter methylation is correlated with improved response to TMZ in glioblastoma patients [2]. However, few systematic studies are available that examine the association between MGMT promoter methylation and MGMT expression in pituitary adenomas [5, 6, 12]. Therefore, the role of promoter methylation in regulating MGMT expression in pituitary adenomas remains controversial. In the study by McCormack et al., only 9 % of the cases (*n* = 46) displayed methylated promoters, although a significant inverse correlation was found between MGMT expression and promoter methylation [7]. This inverse relationship between MGMT expression and promoter methylation was also observed here in all ten tumors (21.7 %), with methylated promoters showing low-level MGMT staining. In addition, MGMT promoter methylation tended to be more common in the aggressive pituitary adenomas, with 33 % in pituitary carcinomas, 42 % in SS3 adenomas, and 43 % in aggressive pituitary adenoma [12, 38]. This is consistent with the fact that the aggressive subtypes are associated with low-level MGMT expression. In contrast, a significant relationship between methylation status and MGMT immunoexpression was not observed in the carcinoma and SS3 pituitary adenomas [12], and a methylated MGMT promoter did not tend to correlate with a response to TMZ exposure [5]. Together, MGMT promoter methylation is likely to explain low-level MGMT expression in some, but not all, pituitary tumors, as further regulation may occur at the transcriptional, post transcriptional, or translational levels.

The tumor suppressor p53 is expressed in response to stress, and plays a central role in pituitary adenoma pathogenesis [25]. Although p53 is widely mutated in many human cancers, it is rarely mutated in pituitary adenomas [25]. In the present study, almost 40 % of the samples had 5 % or more positive staining of p53, which was negatively associated with MGMT immunoexpression, as has been confirmed in other human cancers, including breast, lung, and pancreatic [15, 16]. Moreover, MGMT suppression was associated with p53 activation, and accumulation of p53 increased the cell response to alkylation agents [17–20]. Additional evidence revealed that wild-type p53 suppresses MGMT by binding directly to the MGMT promoter or by sequestering Sp1 [9]. On the contrary, some reports suggest that p53 may positively regulate MGMT expression, and that p53 inhibition sensitizes human glioma cells to TMZ [10, 21]. Expression of wild-type p53 was necessary for inducing MGMT mRNA and protein by ionizing radiation [18]. Additionally, wild-type p53 rather than mutant p53 promoted MGMT expression by modulating MGMT methylation, presumably by reducing DNMT1 (DNA-methyltransferase 1) expression in lung cancer cell lines [22].

Our study has several limitations. First, the number of specimens for evaluating promoter methylation was relatively small, which may fail to describe the complete picture of prolactinoma MGMT promoter methylation and its correlation with tumor and patient characteristics. Second, because of insufficient tumor tissue, we failed to detect MGMT expression by western blot, but relied solely on immunohistochemistry staining. The lack of standard scoring, and operator variability, made it more difficult to determine accurately the level of MGMT expression. Therefore, three independent observers assessed immunoreactivity, with the data analyzed using the most widely used methods. Third, patients in China have no excess to cabergoline. Some patients included in the study may have been treated with cabergoline and not needed surgery or temozolomide treatment. Moreover, these data are not applicable to all prolactinoma patients, but only to the type of specimens analyzed here. Thus, it is difficult to say whether MGMT reduced expression was a general characteristic of prolactinomas. In addition, because all patients were not treated with TMZ before or after surgery, a possible relationship between MGMT level and TMZ intolerance could not be established. Finally, because of the nature of retrospective studies, the causality of MGMT silencing and promoter methylation or p53 protein activation could not be established. Although an inverse correlation between p53 and MGMT was found, it was weak. Nevertheless, our results strongly suggest that promoter methylation is not the sole mechanism underlining MGMT silencing in prolactinomas.

## Conclusions

The present study is one of the largest reports describing prolactinoma MGMT expression and its relationship with patient and tumor characteristics. The prevalence of low-level MGMT expression in prolactinomas, especially in atypical prolactinomas, was confirmed, rationalizing the utility of TMZ as an alternative prolactinoma treatment. Furthermore, this study is the first to show that MGMT expression was inversely correlated with both promoter methylation and p53 protein, implying an epigenetic and transcriptional modulation in silencing MGMT expression.

## Abbreviations

MGMT: O^6^ methylguanine-DNA-methyl transferase
TMZ: Temozolomide
sp1: Specificity protein 1
PRL: Prolactin
GH: Growth hormone
MSP: Methylation-specific PCR, CI, confidence interval
